# Premature Senility

**Published:** 1902-01-25

**Authors:** 


### PREMATURE SENILITY.

To be senile before one's time is a serious affair, and this is what is meant by the term "pre-senility" ; hence, if the symptoms which have been given by some as indicating this condition always had that meaning, they would have to be regarded with great anxiety. Fortunately, however, many of these symptoms, although they are sometimes warnings of premature ageing, are often the results of merely temporary derangements. Bearing this in mind, and remembering that it is in many cases by their association rather than by their individual presence that the meaning of the signs in question are to be discovered, let us run over the list of the neurotic indications of pre-senility as given by Dr. Allan McLane Hamilton,[1] clinical professor of medicine in the Connell University Medical College. After speaking of the changes discoverable by the sphygmograph, he says that the condition of cerebral irritability, in which flushes of hyperæmia occur in an anæmic brain, is an indication of pre-senility. Under slight emotional stimulation such patients become flushed and irascible, or lose the patience and ability to throw off care which may have been characteristic of former years. Hypochrondriasis, too, is a development of that introspection which accompanies a limitation of mental power and easily produced brain fag.

Another early indication of mental failure is an indisposition to make intellectual exertion and a tendency to avoid anything that may be regarded as complex reasoning. If such a person be a professional man he creates little or no new work, and, "under all circumstances, he seeks the broad road of colloquialism and thoughtless speech." "An early impairment of memory, especially of substantitives, annoys the subject," little mistakes are made in calculations, letters are misdirected and cheques are unsigned.

Many men between 30 and 40, who have enjoyed good health and have clear brains, are apt under stress of hard work to suffer from headaches of a peculiar kind which are undoubtedly connected with alterations in the vascular supply. The pain is sub-occipital or frontal and usually bi-lateral, thus differing from migraine. Such attacks nearly always follow fatigue, especially mental fatigue. There is pallor, small pulse, subjective coldness, loss of appetite, emotional depression, and copious discharge of large quantities of limpid urine of low specific gravity. During the attack there is sometimes hemiopia, and there are various other perverted sensations —weight and tiredness in the lower extremities, and hyperæsthesia leading to discomfort from the pressure of the clothes, with, sometimes, cramps in the lower limbs.

Vertigo is not infrequently a symptom of progressive arterial occlusion. As quite an early symptom, the pre-senile individual is apt under unusual cardiac stimulation to become giddy. This may occur from nothing more than an excited dispute or from temporary exposure to extreme heat or cold, or from so small a thing as bending over to tie one's shoe, any of which may easily cause confusion and slight tottering.

Pre-senile insomnia is usually dependent upon an irritable brain. The patient is exhausted toward the latter part of the day, seeks his bed soon after dinner, and after a short sleep awakes, or does so at a very early hour of the morning. In both cases the awakening is sudden and complete, and he is tortured by a veritable kaleidoscope of active thoughts. The pre-senile person, again, is likely to be influenced greatly by excesses in diet, or by indulgence in alcohol or tobacco; to be influenced, that is, far more than has been his wont in times gone by. Perhaps we may add that while the persistent repetition of such symptoms as have just been mentioned must often suggest the oncoming of those vascular changes which are attributed by many to what is described as a "strenuous" life—a life in which work, worry, pleasure, food and drink, may all in turn have been taken in excess—and may thus really indicate a premature ageing or senility, there is hardly one of them the occasional occurrence of which may not be but an accompaniment of that condition which in common parlance is spoken of as "liver," and is in common practice rectified by the old-fashioned blue pill and black draught. Hence it is rather by the grouping of these symptoms, their association with others, and their persistence and tendency to recur from small causes, that they become important as indications of the oncoming of age.

[1] New York Med. Rec., Dec. 28.

